# Near-Field Communication Tag for Colorimetric Glutathione Determination with a Paper-Based Microfluidic Device

**DOI:** 10.3390/bios13020267

**Published:** 2023-02-13

**Authors:** Inmaculada Ortiz-Gómez, Almudena Rivadeneyra, José F. Salmerón, Ignacio de Orbe-Payá, Diego P. Morales, Luis Fermín Capitán-Vallvey, Alfonso Salinas-Castillo

**Affiliations:** 1 ECsens, Department of Analytical Chemistry, Faculty of Sciences, University of Granada, 18071 Granada, Spain; 2 Unit of Excellence in Chemistry Applied to Biomedicine and the Environment, Faculty of Science, University of Granada, 18071 Granada, Spain; 3 Electronic Devices Research Group, Department of Electronics and Computer Technology, Faculty of Sciences, University of Granada, 18071 Granada, Spain

**Keywords:** µPAD, NFC tag, smartphone, colorimetric assay, glutathione determination, health prognosis

## Abstract

Here, we propose a microfluidic paper-based analytical device (µPAD) implemented with a near-field communication (NFC) tag as a portable, simple and fast colorimetric method for glutathione (GSH) determination. The proposed method was based on the fact that Ag^+^ could oxidize 3,3′,5,5′-tetramethylbenzidine (TMB) into oxidized blue TMB. Thus, the presence of GSH could cause the reduction of oxidized TMB, which resulted in a blue color fading. Based on this finding, we developed a method for the colorimetric determination of GSH using a smartphone. A µPAD implemented with the NFC tag allowed the harvesting of energy from a smartphone to activate the LED that allows the capture of a photograph of the µPAD by the smartphone. The integration between electronic interfaces into the hardware of digital image capture served as a means for quantitation. Importantly, this new method shows a low detection limit of 1.0 µM. Therefore, the most important features of this non-enzymatic method are high sensitivity and a simple, fast, portable and low-cost determination of GSH in just 20 min using a colorimetric signal.

## 1. Introduction

Currently, the use of microfluidic paper-based analytical devices (μPAD) has shown high potential to be used in a variety of bioanalytical studies with sensing and diagnostic applications [1]. The attractiveness of the paper in this domain relies on its natural intrinsic porous structure, which is a physical characteristic that enables the paper for spontaneous capillary-based fluidic transport of samples and reagents. This provides multiple benefits, such as reagent storage, mixing, flow control and complex analysis. On the other hand, paper-based devices are powerful detection platforms due to paper material showing high portability and multifunctionality [2,3,4,5]. For all these reasons, µPADs have emerged as a leading alternative for the development of portable and disposable diagnostic devices [6,7,8].

The growing demand for low-cost and in situ monitoring of different chemical analytes in different areas, such as healthcare, food safety and environmental security, demonstrates the need for more reliable and low-power devices that are compatible with wireless communications systems. Another technology that has recently gained much interest is wireless technology based on open standards, which has pushed current technologies towards wireless chemical sensor networks. These technologies include some very well-known global systems for mobile communications, such as Bluetooth, Wi-Fi, radio-frequency identification (RFID) and near-field communications (NFC).

RFID and NFC are interesting short-range radio technologies for their integration with chemical sensors due to their low implementation costs, relative low-level complexity and ultra-low energy/power consumption. Furthermore, they are a convenient substitute for optical detection modalities, which can bring important advantages over other methods of detection in chemical sensing due to their inherent characteristics. RFID and NFC are technologies that are easily miniaturized, flexible, non-destructive, low-cost and passive devices. Furthermore, they can get rid of the batteries or wired connections with external equipment. They realize wireless power and data transmission through electromagnetic induction with the widely used smartphone and are especially compatible with optoelectronic components, light-emitting diodes (LEDs) and photodiodes. In addition, the development of sensing devices based on NFC is a promising approach for on-site detection analysis and wireless monitoring in an enclosed environment because NFC sensors enable smartphones to rapidly obtain sensing data by reading an NFC tag without any physical connection. In fact, NFC sensors provide novel functions for portable, real-time monitoring and quantification, whose advantages have been shown to be very helpful in different areas of healthcare.

A smart, low-cost electronic identification (ID) tag attached to a product allows the product to be identified from a certain distance. By adding more capabilities to the ID tag, the tagged product becomes a smart object [9]. Typically, RFID technology is classified according to the working frequency [10,11,12]. For example, in the frequency band of 13.56 MHz, we can find the NFC protocol designed for interactions between electronic devices in close proximity (<10 cm) [13], which has the attractiveness of being implemented in many mobile devices, such as tablets and smartphones. The latter reduces the complexity of the traditional RFID readers, where it is necessary to have not only a specific reader but also a computer application to extract and process the read data. In this regard, we can take advantage of this technology and use a nonspecific device to read a passive RFID tag with sensing capabilities by only installing a mobile application to access all these functions. In this line, Kassal et al. [14] showed a wireless smart tag for ultra-low power chemical sensing. In particular, this tag autonomously measures the electrode potential and stores this value in its internal memory. Then, these logged data can be wirelessly transferred to an RFID reader or a smartphone through NFC. Another example showed a wireless blood glucose measurement device, which used the 13.56 MHz magnetic field provided by the NFC interface to provide the electric energy required to power all electronic components of the system [15]. Other examples based on this technology have demonstrated its capability to colorimetrically detect glucose, H_2_O_2_ [16] and gases such as O_2_, CO_2_ and NH_3_ and relative humidity concentrations [17], the pH [18] and fruit analysis [19]. Additional examples include strain [17] and temperature [18] monitoring, among others. Therefore, here, we present a passive NFC tag for chemical detection. This tag comprises a coil inductor as the antenna and an RFID chip compatible with the NFC protocol.

We have included a light emitter diode (LED) in this basic RFID tag, which was powered up by the chip’s internal rectifier. As a result, we provide a portable and battery-less illuminant source for the colorimetric measurement of glutathione (GSH). Moreover, the simplicity of the electronics in the NFC tag guarantees its cost-effectiveness and low-power consumption as compared to other solutions from the literature.

GSH is the most abundant biothiol in mammalian cells and plays an important role in redox homeostasis cells, in the nervous system, as a redox modulator of ionotropic receptor activity and as a neurotransmitter [20]. Additionally, GSH plays key roles in biological systems by supporting many cellular functions, being also an important biomarker related to many diseases, such as cancer, liver disease, heart disease and psoriasis, among others. Thus, monitoring the deficiency or increase in GSH levels is critical for disease diagnosis and management. Nowadays, different analytical tools are used for the determination of GSH, such as high-performance liquid chromatography (HPLC) [21] and mass spectrometry [22]. Although most of these methods have high sensitivity, they have some disadvantages, such as high costs, time-consuming sample pretreatments and sophisticated instrument manipulation. Recently, there has been growing interest in the development of point-of-care device testing (POCT) for its low cost and simple and fast response based on colorimetric and fluorescent measurements for the determination of GSH [23,24,25,26,27]. However, the development of these probes is limited for the lack of selectivity, despite possessing excellent optical performances. As a consequence, there is an urgent need to develop novel and simpler GSH determination methods.

Hence, it is necessary to develop alternative detection strategies to overcome these drawbacks. Accordingly, we propose a simple colorimetric device for the easy detection of glutation in biological samples using a microfluidic paper-based device and NFC tag implemented with a white LED to be used as an optical source readable by a smartphone. Similarly, an application for the Android operating system has been developed for the power supply and data reception from the NFC tag. The colorimetric probe is based on the fact that Ag (I) serves as an oxidant for 3,3′,5,5′-tetramethylbenzidine (TMB), changing its color to blue. However, the presence of GSH can reduce oxidized TMB along with a complexation reaction with Ag (I), resulting in a blue color fading [28]. The mechanism to extract the signal related to the GSH comprises taking a photograph of the detection area of the µPAD illuminated with a LED activated by the NFC tag. This is a simple system to easily extract colorimetric responses by using a cheap and general-purpose device.

## 2. Materials and Methods

### 2.1. Reagents and Materials

All reagents used were of analytical reagent grade unless stated otherwise and were obtained from Sigma-Aldrich (Sigma-Aldrich Química S.A., Madrid, Spain). All aqueous solutions were made using reverse osmosis-type quality water, Milli-RO 12 plus Milli-Q station from Millipore, with a conductivity of 18.2 MΩ·cm (Millipore, Bedford, MA, USA). 3,3′,5,5′-tetramethylbenzidine (TMB) (CAS No, 54827-17-7); acetic acid (CAS No, 64-19-7); sodium acetate (CAS No, 127-09-3); ethanol (CAS No, 64-17-5); L-glutathione (GSH) (CAS No, 70-18-8); L-cysteine (Cys) (CAS No, 7048-04-6); L-glutamine (Gln) (CAS No, 56-85-9); L-arginine (Arg) (CAS No, 74-79-3); L-asparagine (Asn) (CAS No, 70-47-3); DL-homocysteine (Hcys) (CAS No, 454-29-5); L-glutamic acid (Glu) (CAS No, 56-86-0); D-mannose (Man) (CAS No, 3458-28-4); galactose (Gal) (CAS No, 137868-52-1); D-lysine (Lys) (CAS No, 923-27-3); L-serine (Ser) (CAS No, 56-45-1); L-histidine (His) (CAS No, 71-00-1); Bovine serum albumin (BSA) (CAS No, 9048-46-8); and Silver nitrate (AgNO_3_) (CAS No, 7761-88-8) were acquired from Merck (Darmstadt, Germany). Filter paper (ref. Whatman 1, qualitative circles 150 mm) from Sigma-Aldrich and 54 × 86 mm transparent sheets with a quality strength protection of 125 microns (Fellowes^®^ pouches, Itaca, NY, USA) were used to prepare and laminate the μPAD.

### 2.2. Instruments and Software

The color change in the μPAD was measured with an LG Nexus 4 smartphone (Android). A Rayjet Trotec Laser engraving printer (Rayjet, Trotec, Barcelona, Spain, commanded by Rayjet Commander Software 1.0, was used to design and fabricate the µPAD. A 3D printer Witbox 2 (Witbox, Pamplona, Spain) was used to fabricate the 3D box (83 × 163 × 75 mm), which was made using polylactic acid (PLA), where the NFC tag, the µPAD and the smartphone were located during the determination of the GSH.

### 2.3. Preparation of µPAD

The devices were produced on a paper sheet by using a craft-cutting technique as a cost-effective, simple and reproducible process [29]. The pattern of μPAD was first designed using Illustrator software (Adobe Systems, San Francisco, CA, USA) and the design was exported as an FS file to the controller software of a desktop Laser engraver with a 12 W CO_2_ laser source. In order to optimize the procedure and the substrate used, the µPAD elements were produced by cutting a sheet of cellulose paper (diameter 185 mm). The rate of success was 99%, confirming the viability of the design.

**Figure 1:**
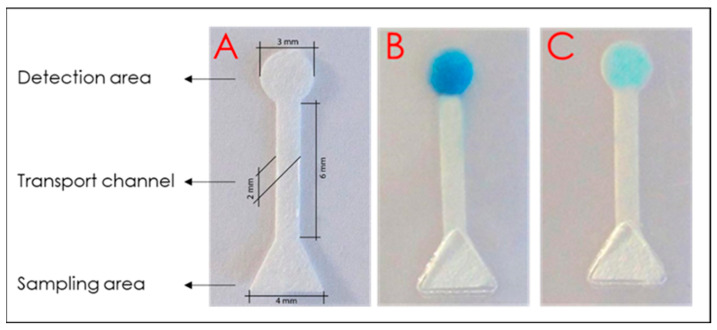
Picture of the µPAD (**A**) after adding a sample without GSH (**B**) and with 200 µM of GSH (**C**). Conditions: 0.5 µL AgNO_3_ 15 mM, 0.5 µL TMB 12.5 mM, 15 µL of sample and 20 min of reaction time.

The device shown in Figure 1A comprises three separate areas, one for sampling, a transport channel where the AgNO_3_ and buffer are spotted, and a detection area where the chromogenic reagent is located. To prepare the µPAD for the colorimetric detection of the GSH, firstly, 0.5 µL of 12.5 mM TMB was added to the detection area. Next, 0.5 µL of the 15 mM AgNO_3_ solution containing 100 µL of acetic acid/acetate buffer (100 mM, pH 4) was added over the transport channel. Then, the µPAD was laminated to protect the sample after its incorporation into the analytical device by means of a manual laminator at a temperature of 150 °C using a thermoplastic material (54 × 86 mm). A hole in the sampling area of the µPAD allowed the sample through the device. After that, the μPADs were stored in a dry environment at 4 °C in the dark until use.

### 2.4. Tag Design and Fabrication

The tag was designed to resonate at the high frequency (HF) band, centered at 13.56 MHz. The operation mode in this frequency band is coupling; thus, the antenna is a coil inductor. The Advanced Design System (ADS) software (Keysight Technologies, Santa Clara, CA, USA) based on the momentum method was used for designing the antennas and for studying their RF electrical behavior, taking into account their surface area roughness and the influence of the substrate [13].

**Figure 2:**
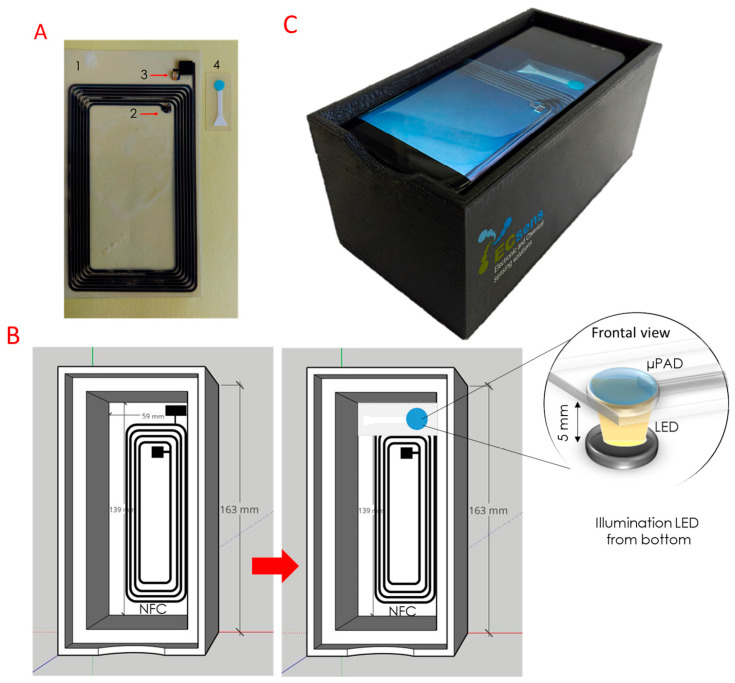
(**A**) Illustration of measurement system: 1. RFID chip; 2. Connection of inner and outer parts of the coil inductor; 3. LED connected to the chip through its VBAT and VSS pins; 4. µPAD. (**B**) Accessory for picture acquisition with the smartphone. (**C**) Scheme of the measurement setup for the colorimetric detection of GSH using the NFC tag and the µPAD.

The SL13a (ASM AG, Unterpremstaetten, Austria) was chosen as the RFID chip. It has a capacitance of approximately 25 pF at f0 = 13.56 MHz. Not considering the parasitic resistance, a value of 5.5 µH for the coil antenna should be achieved to enable the resonance at the desired frequency, avoiding the inclusion of an external capacitor [13]. This chip has an inbuilt temperature sensor with a nonlinearity of ±0.5 °C with a maximum range from −40 °C to 74 °C. A 10-bit analog-to-digital converter (ADC) and an 8 kbit EEPROM are included in the chip. Two different white LEDs have been tested in order to study the influence of the light source on the system. For this purpose, the NSPW310DS (Nichia Corporation, Tokushima, Japan) and SMLP12WB (ROHM Semiconductor, San Diego, CA, USA) were selected. The former has a circular section with a diameter of 3.5 mm, whereas the latter has a diameter of 0.6 mm. Figure 2 presents the layout of this RFID tag. In order to provide the chemical sensing capabilities, we have included a light emitter diode (LED) between the VDD and VSS pins of this chip. A serial resistor has been added to polarize the LED.

This tag has been manufactured with a milling machine (Cirqdroid, Riga, Latvia) on an FR-4 substrate. Accuracy in fabrication plays an important role in RF circuits; therefore, specific RF tools were employed in the milling process. The placement and soldering of the components were carried out by using an infrared solder station, model IR/PL 550 (Kurtz Holding GmbH & Co., Kreuzwertheim, Germany).

In order to study the influence of the reader on the read range and LED power, three different commercial RFID readers were used to test the system. In particular, an HF TRF7960 (Texas Instruments, Dallas, TX, USA) compatible with ISO 15693, a TRF7970 (Texas Instruments, Dallas, TX, USA) compatible with ISO 15693 and NFC protocol, and a smartphone LG Nexus 4 (Google, Los Angeles, CA, USA) compatible with NFC were employed in this work. The RFID readers power up and drive the tags using commands of the different protocols. The characterization of the coil inductor parameters was carried out with an impedance analyzer E42990A and an impedance probe kit (42941A) (Keysight Technologies., Santa Clara, CA, USA) by measuring its inductance and quality factor, using the four-wire measurement technique and using an excitation voltage of VDC = 0 and VAC = 500 mV.

### 2.5. Measurement Protocol

For the GSH determination, 10 μL of standards or problem samples containing GSH were dropped in the sampling area of the microfluidic device. After that, the solution flowed by capillarity towards the zones where the reagents were placed. Twenty minutes are needed to complete the reaction in the detection zone (Figure 1C). Once the sensing area was reacted, a picture of the microfluidic device was captured using an accessory made with a 3D printer and a smartphone (Figure 2). The GSH concentration was calculated from the calibration function obtained with the GSH standards.

The mechanism to extract the measurement is to locate the detection area of the µPAD onto the LED. Then, the NFC-capable smartphone that is in close proximity to the tag sends enough power to the tag to turn on the LED. Our proposed method uses a white LED to avoid environmental light influence since the LED produces strong and steady light.

The detection area of the µPAD changes its colorimetric response related to the concentration of the GSH present in the sample. A photograph of the detection area of the µPAD is captured by the smartphone and later processed to extract the S parameter or saturation value of HSV color space, which is employed as the analytical parameter. Once the saturation value is processed, it is shown on the mobile display and stored in the smartphone’s memory.

### 2.6. Detection of Glutathione in Real Samples

A colorimetric assay of the GSH in serum samples from healthy humans was carried out as follows. First, serum samples from local hospitals were spiked with different amounts of GSH, filtered by 0.22 µm and 100-fold diluted by purified water. After that, 10 μL of each sample was placed in the sampling area of the microfluidic device, and after 20 min, a picture was taken. Although this procedure can be conducted with any HF RFID reader, we consider that using NFC benefits the system because anyone can use this system with just a smart terminal with NFC capabilities without requiring a specific device to perform the measurement.

### 2.7. Mobile Application

The first step before the development of a smartphone application is the selection of the operative system in which the application will run. In our case, Android was chosen as the operating system not only because of the ease of programming and free license against other proprietary operating systems, such as iOS (Apple Incorporation, Los Angeles, CA, USA) or BlackBerry OS (BlackBerry, Mississauga, ON, Canada) but also because the vast majority of NFC terminals are supported in Android. [9]. Furthermore, our application requires the use of the NFC protocol, and the number of Android devices incorporating this technology is the largest.

In the current work, an LG Nexus 4 (Google, Los Angeles, CA, USA) was selected as the detection platform. This smartphone has a quad-core processor running Android version 4.4.4. The phone has a display of 470.07 inches with 1280 × 768 pixels of resolution with total dimensions of 133.9 mm × 68.7 mm × 9.1 mm. This terminal has an inbuilt camera of 8 megapixels.

Following the same procedure as in [16], the camera parameters were fixed in the developed application in order to avoid automatic configurations of the smartphone. In addition, the application uses the autofocus option, which avoids capturing blurred and out-of-focus pictures of the microfluidic device. In order to use a different smartphone, the focal distance of the smartphone’s camera with respect to the detection zone of the µPAD should be calibrated first.

The resolution of the pictures was set to the highest available value in this phone, which is 3264 × 2448 pixels, in order to guarantee a high number of pixels for the analysis of the image. Despite the higher resolution, the time of processing was also increased for this resolution. All pictures were saved in Joint Photographic Experts Group (JPEG) format, which is the default format in most of the smart terminals, with a resolution of 96 dots per inch (dpi) and 8 bits per RGB channel. During the acquisition of the picture, the smartphone needs to be maintained in a parallel position with the RFID tag in order to reduce distortions in the shape of the sensing areas due to the inclination of the mobile device (Figure 2B). Furthermore, the smartphone must be close enough to the RFID tag to provide enough energy to power up the LED. Thus, if the smartphone is not correctly placed, the LED does not provide emission. In addition, if a different smartphone is used in this system, calibration in the reading distance to power the LED might be required.

In the case of smartphones with NFC capabilities, once this feature is activated, every time the NFC tag or system is placed in the read range of a smartphone, the phone tries to establish communication with the other device via the NFC protocol. Therefore, it is not necessary to include any NFC code to power the LED in the application unless we want to extract the ID, temperature value or other external connected sensors. Nevertheless, we programmed the app to extract the tag ID also as well as the time and date when the measurement was performed so that all these parameters, together with the HSV coordinates, could be stored in an internal database.

## 3. Results

### 3.1. Mechanism of GSH Detection

**Scheme 1:**
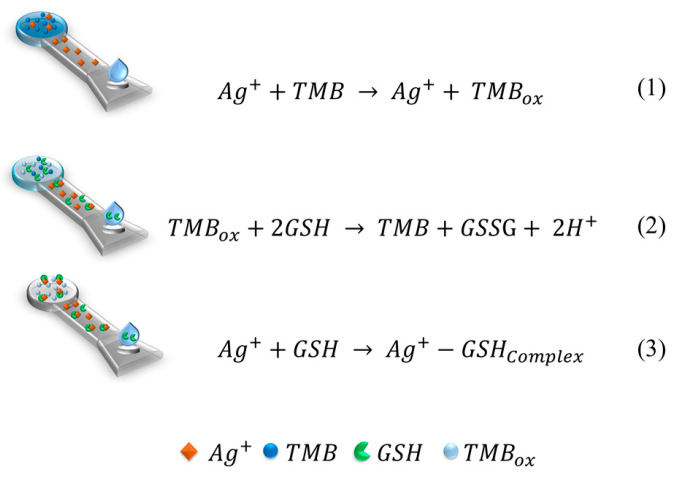
Illustration of the mechanism for GSH determination.

**Figure 3:**
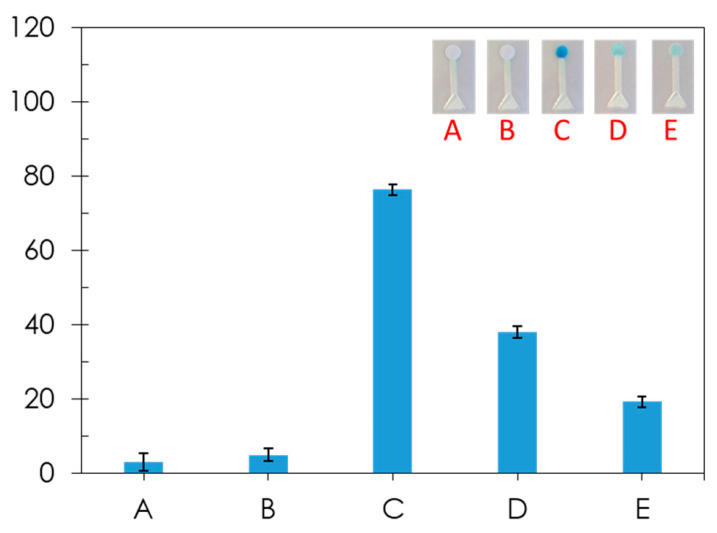
Response of the µPAD to AgNO_3_ (A), TMB (B), Ag^+^ + TMB (C), Ag^+^ + TMB + GSH 150 µM (D) and Ag^+^ + TMB + GSH 250 µM (E). The insets show the corresponding photography images of the µPAD in each case. Error bars represent standard deviations from three different µPADs.

The GSH detection mechanism is shown in Scheme 1. Previous research [30] described that Ag^+^ could directly oxidize TMB to the oxidized TMB (Equation (1): A^+^ + TMB → Ag^+^ + TMB_ox_) and induce a blue color change. Subsequently, the presence of GSH in the Ag^+^-TMB system caused the reduction of oxidized TMB to form colorless TMB (Equation (2): TMB_OX_ + 2GSH → TMB + GSSG + 2H^+^), which resulted in the fading of the blue color. On the other hand, GSH reacts with Ag^+^, forming a complex (Equation (3): A^+^ + GSH → Ag^+^-GSH_complex_), both of which result in a blue color fading. In order to verify this mechanism, controlled testing was carried out at room temperature using 0.5 µL of 12.5 mM of TMB, 0.5 µL of 15 mM AgNO_3_ in 100 µL of acetic acid/acetate buffer 100 mM (pH 4) with 10 µL of different concentrations of GSH. The results, displayed in Figure 3, show that the reagents (AgNO_3_ and TMB) do not generate color changes in the µPAD. Thus, the blue color in the detection area of the µPAD is strong and obvious in the TMB plus Ag^+^ system after adding 10 µL of a sample without GSH, owing to the formation of TMBox. In addition, the detection area of the µPAD changes the blue saturation when 10 µL of 150 µM and 250 µM of GSH were added.

These results confirmed that TMBox could be reduced by GSH, resulting in a decrease of the saturation coordinate in the detection zone of the µPAD. Thus, a straightforward method for GSH determination could be established by using the Ag^+^-TMB system as a colorimetric assay.

### 3.2. Optimization of GSH Determination Conditions

**Figure 4:**
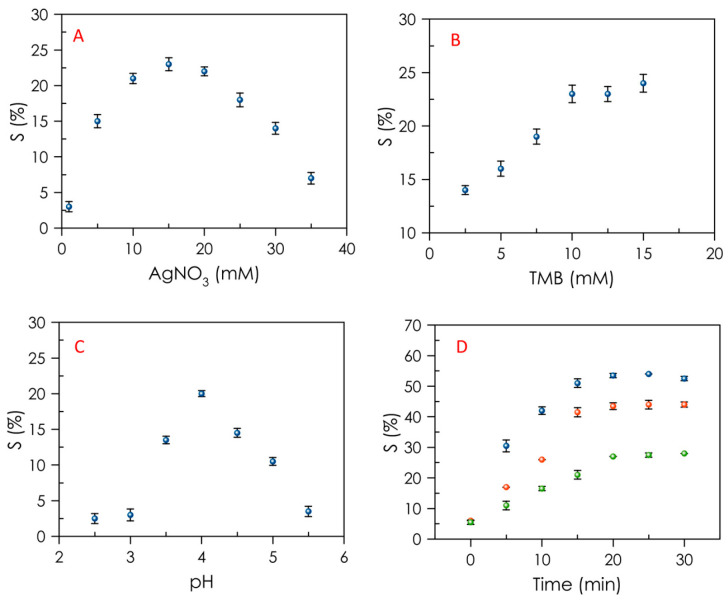
Optimization of the reaction conditions for GSH detection: (**A**) AgNO_3_ concentration. Conditions: 0.5 µL AgNO_3_ at different concentrations, 0.5 µL TMB 15 mM, 15 µL GSH 200 µM; (**B**) TMB concentration. Conditions: 0.5 µL AgNO_3_ 12.5 mM, 0.5 µL TMB at different concentrations, 15 µL GSH 200 µM; (**C**) pH dependence on GSH recognition. Conditions: 0.5 µL AgNO_3_ 12.5 mM, 0.5 µL TMB 15 mM, 15 µL GSH 200 µM; and (**D**) reaction time for 50 µM (blue dots), 150 µM (red dots) and 250 µM (green dots) of GSH. Conditions: 0.5 µL AgNO_3_ 15 mM, 0.5 µL TMB 12.5 mM. Error bars represent standard deviations from three µPADs.

Several aspects that affect the sensitivity of this method to detect the GSH were optimized, such as the concentration of Ag^+^ and TMB, the pH value of the buffer and reaction time (Figure 4).

In order to study the effect of the concentration of added Ag^+^ in the µPAD, 0.5 µL of the AgNO_3_ solution, at concentrations between 1.0 mM to 35 mM, were added to the transport channel (Figure 4A). It can be clearly observed that the concentration of AgNO_3_ being too high or too low is not suitable for GSH detection because the saturation value is too low. If the concentration of AgNO_3_ is higher than 15 mM, the saturation value decreases. This occurs because the AgNO_3_ forms a layer onto the transport channel, and the sample cannot go through the channel to the detection area where the TMB is located. Similarly, if the concentration of AgNO_3_ is too low, the saturation values are too low, and the detection of glutathione is not possible. According to the results, we decided to fix the concentration of AgNO_3_ at 15 mM.

The effect of the concentration of TMB is shown in Figure 4B. The reagent TMB is placed into the detection area of the µPAD from an ethanolic solution due to its high solubility and evaporation rate in this solvent. The optimal concentration of TMB was studied in separate experiments where we were preparing devices by adding a drop casting of 0.5 µL of a TMB solution between 2.5 mM and 15 mM. The results showed that when the TMB concentration increased, the color of the detection zone on the µPAD exhibited a significant saturation change of up to 12.5 mM. High saturation values are needed to increase the sensitivity. Thus, the selected concentration considered optimal for TMB was 12.5 mM.

The impact of the pH value in GSH determination was investigated since the pH has an important effect on the charge properties of amine, carboxyl and thiol groups of GSH [30,31]. In addition, it was reported that the effect of the pH had an effect on the charge of some groups (-NH2, -COOH and -SH) of GSH; thus, extreme pH values were not suitable for this reaction. The pH was adjusted by placing a buffer into the transport channel of the device along with the silver nitrate solution. As shown in Figure 4C, the influence of the pH was investigated in a pH ranging from 2.5 to 5.5 using 100 mM of acetic acid/acetate buffer. An increase in pH from 2.5 to 4 resulted in an increased saturation value for the oxidation of TMB in the detection zone in the µPAD due to the high catalyst activity of the Ag (I) at pH 4. These results are in agreement with those obtained by other studies [30,32]. Furthermore, the saturation value decreased when pH values were above 4. As a consequence, pH 4 was defined as being the optimal pH for GSH determination.

The reaction time was studied by taking photos of the µPAD for 30 min, as shown in Figure 4D. Thus, the results indicated that after 20 min of reaction, the saturation of the detection zone was constant due to the µPAD being totally dry. In order to double-check that the µPAD was totally dry, it was weighed before and 20 min after loading the sample. Initially, the µPAD weighed 16 mg, and 20 min after loading 10 µL of the sample, the µPAD weighed 16 mg. Consequently, 20 min was selected because it was sufficient to complete the reaction and dry the paper to obtain the picture.

### 3.3. Characterization of the Tag Acquisition System

The operation of the LED is limited by the maximum available power from the rectifier of the RFID chip. In the case of the SL13A, the maximum voltage is 3 V and the maximum current is 4 mA; therefore, the ideal maximum available power is 12 mW.

Both LEDs have been chosen to work with a forward voltage as close as possible to 3 V, taking into account the RFID chip power constraints. According to the manufacturer information for the maximum available current (4 mA), a forward voltage of about 2.6 V and 2.9 V can be achieved for the 3 mm LED and SMD LED, respectively. When the tag with the 3 mm LED was placed at a 10 mm distance from the reader, the polarization I-V point was 0.15 mA–1.66 V, leading to a power consumption of 0.25 mW. This gave us a luminance of 1500 mcd, according to the manufacturer. In a similar way, the SMD LED tag at the same distance of 10 mm operated at 1.60 V and 0.14 mA. The power consumption was 0.21 mW, and the associated luminance was 11 mcd.

**Figure 5:**
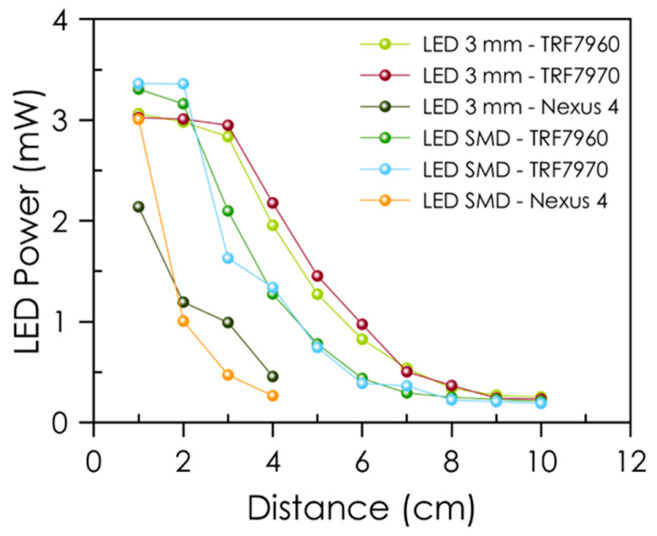
LED polarization according to the distance between the tag and the reader.

The larger the distance between the reader and the tag, the lower the amount of power rectified by the chip, leading to a lower LED luminance. Figure 5 shows both of the LED operating points using three different commercial readers. There was a limited distance where the associated rectified power and luminance were not enough to capture the sensor information. In our case, the maximum distance was 8.0 cm for the TRF7960/7970 readers and 4.0 cm for the LG Nexus 4 reader. The highest read range was achieved with the TRF7960, and the lowest range with the smartphone. This result can be explained by the fact that the mobile terminal has a smaller antenna than the other two commercial readers.

**Figure 6:**
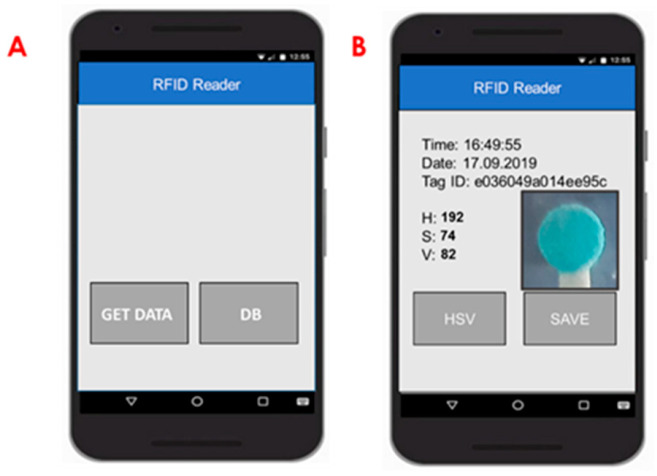
(**A**) Visual representation of home screen; and (**B**) selecting area of analysis. Conditions: 0.5 µL AgNO_3_ 15 mM, 0.5 µL TMB 12.5 mM, 15 µL of sample, 20 min of reaction time.

The measurement setup comprises the developed µPAD, the designed NFC tag with the LED and the smartphone with the Android application (see Figure 6). Once the app was initialized, the first step was to check if the NFC was enabled in the smartphone. If so, the user could select to obtain data or go to the database (DB) (Figure 6A). Once we are in the “get data menu” and place the phone next to the tag, we can see the ID, time and date (Figure 6B). Then, in order to acquire the HSV coordinates, we press that button and are redirected to the phone camera to take a picture (Figure 6B). Once we save the picture, we can select the area to be analyzed with our fingertip (Figure 6B). If the user presses “OK”, it will now redirect back to the former screen with both NFC and HSV data taken. All this information can be stored in the internal database by clicking on the “save” button (Figure 6B).

The electronic system, together with the mobile application described in this work, can be used in other colorimetric measurements by modifying the equation that relates the HSV coordinates to the parameter of interest. Moreover, other colorimetric coordinate spaces, such as CIELab, XYZ and RGB, could be used as well. This opens a wide range of applications for the built platform.

### 3.4. Analytical Characterization

**Figure 7:**
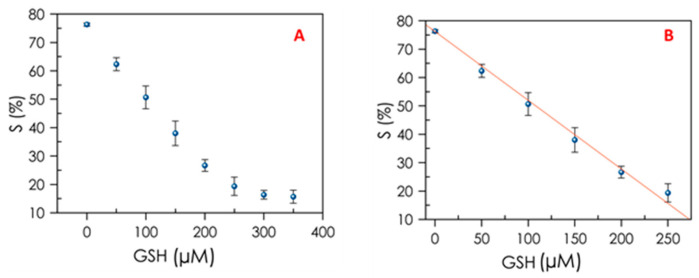
(**A**) Dependence of GHS concentration obtained with the smartphone and (**B**) using the calibration function. Conditions: 0.5 µL AgNO_3_ 15 mM, 0.5 µL TMB 12.5 mM, 15 µL GSH standard at different concentrations, 20 min of reaction time. Error bars represent standard deviations from three µPADs.

Based on the relationship between the change in saturation coordinate S of the blue color of the detection zone in the device and the GSH concentration, a sensitive colorimetric assay based on paper for GSH detection was established. This monotonal color change was followed by the saturation coordinate S of the HSV color space after imaging the device with a smartphone. The S value decreased with GHS concentration, as shown in Figure 7. The analytical function was obtained through a calibration set formed by nine GSH standards between 50 and 400 µM, including three replicates of each one and using 20 min as the reaction time.

The experimental dependence between the S value and GSH concentration obtained with a smartphone was linear. The regression equation was S = −0.2C + 74.9 with R^2^ = 0.9965. The limit of detection (LOD) GSH was calculated in light of the 3σ/slope laws to be as low as 1.0 µM. In addition, the limit of quantification (LOQ) was calculated by the 10σ/slope equation, resulting in 3.0 µM, which was an acceptable value when taking into account the normal values found in biological fluids. The reproducibility of the proposed method, as RSD, was demonstrated by six different µPADs working at 200 µM glutathione and was 2.3%, an acceptable precision considering the measuring system used. The cost of the µPAD, considering only the reagents and the paper for the device, was around 0.05 cents/unit. The performance characteristics of the µPAD developed are presented in Table 1. In order to evaluate the stability of the developed µPAD, eighteen µPADs were stored in the dark at 4 °C under an inert atmosphere (nitrogen). Every five days, six µPADs were removed from storage, three were tested with a sample without GSH, and three were tested with 100 µM of GSH solution, showing good stability during this time. The µPADs tested with GSH 100 µM showed a saturation value (%) of 50 ± 2.0 in the first week, 51 ± 1.5 in the second week and 49.5 ± 2.1 in the last week, and the samples without GSH showed a saturation (%) of 72 ± 2.6 in the first week, 73 ± 2.1 in the second week and 73 ± 2.5 in the third week.

A comparative study of the characteristics from different colorimetric procedures for GSH is presented in Table 2, showing that the µPAD described had a wide range and good detection limit, as compared to other devices. As also shown in Table 2, the linear range of our method is wider than or comparable to some of them. The proposed method was therefore providing a convenient and efficient detection device for GSH determination. The detection limit obtained in our method was lower in comparison with others. Hence, our method provides a suitable alternative for GSH determination in real samples. Moreover, the detection time was much shorter than in many previous reports. In summary, we concluded that our method was feasible, fast and cost-effective.

## 4. Interferences

**Figure 8:**
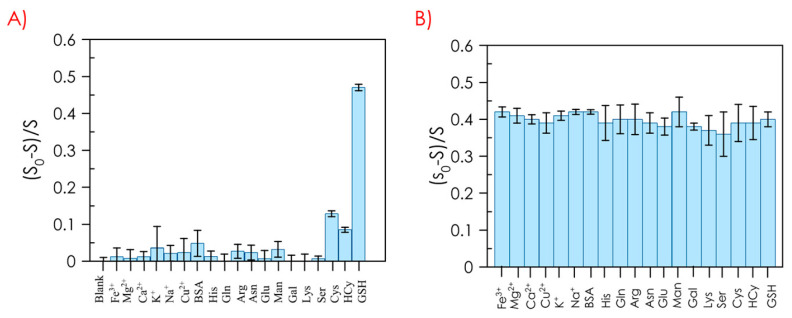
Selectivity of the µPAD towards GSH without (**A**) and with (**B**) GSH. Real samples analysis: Conditions: 0.5 µL AgNO_3_ 15 mM, 0.5 µL TMB 12.5 mM, 15 µL sample 100 µM, 20 min of reaction time. Error bars represent standard deviations from three µPADs.

In order to evaluate the selectivity of the proposed method, we examined the influence of some potential interferences in serum, including Fe^3+^, Mg^2+^, Ca^2+^, K^+^, Na^+^, Cu^2+^, BSA, Cys, Hcy, Lys, Ser, His, Gln, Arg, Asn, Glu, mannose and galactose. Solutions containing 100 µM GSH and 100 µM interference were analyzed in triplicate (Figure 8). The reaction conditions were the same as those described previously. The difference in the saturation values between the blank and interferences was normalized, where S0 was the saturation value of the blank, and S was the saturation value for each interference. Even though the results showed that Cys and Hcy induced some small colorimetric changes, they were lower than the GSH, similar to previously published studies [30,31,39]. However, the presence of GSH masked the effect of the colorimetric changes in Cys and Hcy [30] (Figure 8B). In addition, the concentrations of Cys, Hcy and GSH varied significantly among different biological fluids (blood, serum, urine) where the prevalence of specific biothiols over others was observed (Cys in urine, Hcy in blood and GSH in serum). Therefore, we found that the potential interferences had negligible effects on GSH detection.

## 5. Real Samples Analysis

In order to verify the potential of the developed µPAD for the quantitative determination of GSH in real samples, the concentration of GSH in human serum samples was studied. It is well-known that the normal level of GSH in human serum is 7–21 µM [40]. Blood samples were obtained from healthy volunteers, and their serum samples were analysed after prior dilution at a 1:100 ratio in water and spiked with different amounts of GSH [41,42]. Afterwards, 10 μL of each sample was placed in the sampling area of the microfluidic device, and after 20 min, a picture was taken. The results of the analysis are given in Table 3. It can be observed that the measured values were consistent with the serum GSH concentration values. The recoveries of different known amounts of added GSH were obtained from 98.3 to 104.5% in the serum samples. Altogether, these results indicated that the proposed method is accurate and sensitive enough for the detection of biothiols in real human samples.

## 6. Conclusions

In summary, in the present work, we have settled a portable, simple and low-cost assay for the colorimetric determination of GSH using a microfluidic paper-based analytical device implemented with the NFC tag and using a smartphone in a 3D accessory. The interaction of the NFC interface with a smartphone allowed for image processing and recording color changes onto the microfluidic paper-based analytical device to translate them into quantitative information, which was obtained by digital image capture and analysis. Thus, our method showed a detection limit of 1.0 µM for the GSH, with a linear dynamic range from 3.0 to 250.0 µM. Moreover, the developed µPAD in this paper can be easily applied for the detection of other analytes and has great potential to be used in different healthcare areas, allowing fast detection, cost-effectively and with high sensitivity analysis by using a very simple operational process.

## Figures and Tables

**Table 1:** Analytical characterization of µPAD for GSH determination.

Analytical Parameters	Smartphone
Measurement range (µM)	3.0–250.0
Slope (µM)	0.2
Intercept (a.u.)	74.9
LOD (µM)	1.0
LOQ (µM)	3.0
Precision (%) 200 µM	2.3

**Table 2:** Comparison of different colorimetric methods for GSH detection.

Materials	Linear Range (µM)	LOD (µM)	Reaction Time (min)	Method	References
Fe^+3^-TMB	2.0–24.0	1.04	10	Solution	[33]
Rhodamine B-gold NPs	12–1384	1.0	60	Solution	[34]
Ag^+^-TMB	0.05–8.0	0.05	10	Solution	[30]
MnO_2_ NPs-TMB	0.26–26	0.1	15	Solution	[35]
Fe_3_O_4_-ABTS-H_2_O_2_	3.0–30	Not given	20	Solution	[36]
Ag:AgCl	10–100	10.0	5	Paper	[37]
Au NPs	8–75	6.9	40	Paper	[38]
Ag^+^-TMB	3.0–250.0	1.0	20	Paper	This work

**Table 3:** Recovery study for GSH in human serum samples.

Concentration of GSH (µM)			
Sample	Amount Added	Amount Found	Recovery (%)
Serum 1	50	54.9	100.9
	200	209.1	104.5
	250	254.9	101.9
Serum 2	50	50.8	101.5
	200	196.6	98.3
	250	254.9	101.9
Serum 3	50	51.2	102.4
	200	197.4	98.7
	250	254.9	101.9

## Data Availability

Data is contained within the article. Additional data not presented in this article is available on request from the corresponding author.
